# Nonclassical Vitamin D Action

**DOI:** 10.3390/nu2040408

**Published:** 2010-03-25

**Authors:** Armin Zittermann, Jan F. Gummert

**Affiliations:** Clinic for Thoracic and Cardiovascular Surgery, Heart and Diabetes Center North Rhine-Westphalia, Ruhr University Bochum, Georgstrasse 11, 32545 Bad Oeynhausen, Germany; Email: herzchirurgie@hdz-nrw.de

**Keywords:** vitamin D, cancer, cardiovascular, mortality, ultraviolet B radiation, diet

## Abstract

It is becoming increasingly clear that vitamin D has a broad range of actions in the human body. Besides its well-known effects on calcium/phosphate homeostasis, vitamin D influences muscle function, cardiovascular homeostasis, nervous function, and the immune response. Vitamin D deficiency/insufficiency has been associated with muscle weakness and a high incidence of various chronic diseases such as cardiovascular disease, cancer, multiple sclerosis, and type 1 and 2 diabetes. Most importantly, low vitamin D status has been found to be an independent predictor of all-cause mortality. Several recent randomized controlled trials support the assumption that vitamin D can improve muscle strength, glucose homeostasis, and cardiovascular risk markers. In addition, vitamin D may reduce cancer incidence and elevated blood pressure. Since the prevalence of vitamin D deficiency/insufficiency is high throughout the world, there is a need to improve vitamin D status in the general adult population. However, the currently recommended daily vitamin D intake of 5-15 µg is too low to achieve an adequate vitamin D status in individuals with only modest skin synthesis. Thus, there is a need to recommend a vitamin D intake that is effective for achieving adequate circulating 25-hydroxyvitamin D concentrations (>75 nmol/L).

## 1. Introduction

Vitamin D has long been known for its effects on calcium and bone metabolism. Severe vitamin D deficiency causes a lack of bone mineralization, which manifests as rickets in children and osteomalacia in adults. There is also accumulating evidence that insufficient vitamin D status contributes to the bone disease osteoporosis. Adequate vitamin D supplementation can reduce the risk of osteoporotic fractures by approximately 20% [1]. However, it is now becoming increasingly clear that vitamin D has a much broader range of actions in the human body than believed before. Its physiological effects are not only limited to bone. Various other chronic diseases that are frequently observed in modern societies are probably at least in part caused by inadequate vitamin D supply. The present article describes the potential clinical relevance of nonclassical vitamin D actions. It refers to randomized, controlled clinical trials (RCTs) or meta-analyses of RCTs whenever it is possible. Results from non-RCTs are also presented in fields where no RCTs are available yet. Although the article primarily refers to the literature of the last four years, some useful older data are also included. Note that this article should provide evidence for nonclassical vitamin D actions. It is not a systemic review of the available literature.

## 2. Vitamin D Metabolism

Vitamin D is unique among vitamins in that humans can produce it themselves in their skin provided they have sufficient exposure to ultraviolet radiation B (290-315 nm). Vitamin D is also found naturally in small amounts in milk and eggs, and in relatively large amounts in fatty fish such as herring and mackerel. Nevertheless, skin synthesis of vitamin D usually contributes 80% to 90% to vitamin D supply in free-living persons. This assumption is based on the fact that in healthy young adults circulating 25(OH)D concentrations usually lie between 30-80 nmol/L [2], dietary vitamin D intake is usually below 5 µg daily [3], and 1 µg vitamin D increases circulating 25(OH)D concentrations by approximately 1-3 nmol/L [4,5]. The exact amount of vitamin D production in human skin depends on the geographic latitude, season, time of day, as well as on the weather conditions (cloudiness), amount of air pollution and surface reflection. In addition, clothing habits, lifestyle, and workplace (e.g., indoor *versus* outdoor), sunscreen use, and sun avoidance practices have a strong impact on vitamin D synthesis. It is also noteworthy that skin type determines a person’s effectiveness in producing vitamin D. The darker the skin is pigmented, the more ultraviolet radiation is absorbed by melanin and the less vitamin D is produced [6,7]. Migrant populations and their descendants often have skin types that do not fit to the ambient ultraviolet environment. To achieve a similar effect on vitamin D production compared to a fair-skinned person, the exposure time to ultraviolet radiation in a dark-skinned person living in Europe or North America must be up to six times longer [8].

Vitamin D can be produced very effectively by humans when ultraviolet radiation B (UVB) from sunlight or artificial sources reaches skin cells. A whole body exposure to UVB radiation of 15 to 20 minutes daily is able to produce up to 250 µg vitamin D (10,000 IU) [9,10]. Once in the circulation, vitamin D is converted by a hepatic hydroxylase into 25-hyroxyvitamin D (25(OH)D). The circulating 25(OH)D level is an indicator of vitamin D status. This level reflects both, ultraviolet exposure and dietary vitamin D intake. As needed, 25(OH)D is converted in the kidney to its active hormonal form 1,25-dihydroxyvitamin D_3_ (calcitriol) in a process which is usually tightly controlled by parathyroid hormone. In spite of this, inadequate vitamin D supply lowers the circulating level of this important hormone [11]. Circulating calcitriol is also adversely affected by a reduced number of viable nephrons, high serum concentrations of fibroblast growth factor-23, and high levels of inflammatory cytokines [12,13]. 

If vitamin D production or intake is low, vitamin D insufficiency or even deficiency is the result. Parathyroid hormone levels start rising at 25(OH)D cutoff levels of 75 nmol/l or lower (Table 1). The following cut-offs are used for different stages of vitamin D inadequacy: <25 nmol/L for deficiency 

(divide by 2.496 to convert into ng/ml), 25-49.9nmol/L for insufficiency, 50-74.9 nmol/L for hypovitaminosis/suboptimal supply. Although there is still some debate on how to classify vitamin D status, the vast majority of vitamin D researchers agree that 25(OH)D levels below 50 nmol/l are insufficient. 

Cellular vitamin D actions are mediated by a membrane-bound and a cytosolic vitamin D receptor (VDR). The VDR is nearly ubiquitously expressed, and almost all cells respond to vitamin D exposure; about 3% of the human genome is regulated, directly and/or indirectly, by the vitamin D endocrine system [14]. Calcitriol is also produced by local 1α-hydroxylases from its precursor 25(OH)D in various extra-renal cells, among them vascular smooth muscle cells, colonocytes, and immune cells such as monocytes, dendritic cells (DCs), and B-lymphocytes [15,16]. Here, calcitriol plays an important paracrine and autocrine role. Uptake of 25(OH)D into extra-renal tissues is reduced in case of low circulating calcitriol levels, e.g., in patients with renal insufficiency [17].

**Table 1:** Vitamin D status classified according to circulating 25-hydroxyvitamin D concentrations [according to reference 18, with modifications according to reference 6].

Stage	25-hydroxyvitamin D (nmol/l)	Clinical/biochemical alterations
Deficiency	<25	Rickets, osteomalacia, myopathy, calcium malabsorption, severe hyperparathyroidism, low calcitriol concentrations, impaired immune and cardiac function?, death
Insufficiency	25 to 49.	Reduced bone mineral density, impaired muscle function, low intestinal calcium absorption rates, elevated PTH levels, slightly reduced calcitriol levels
Hypovitaminosis D/suboptimal supply	50 to 74.9	Low bodily stores of vitamin D, slightly elevated PTH levels
Adequacy	75 to 372	No disturbances of vitamin D-dependent functions
Intoxication	>372	Intestinal calcium hyperabsorption, hypercalcemia, soft tissue calcification, death

Abbreviation: PTH, parathyroid hormone

## 3. Worldwide Vitamin D Status

A recent review [19] summarized human vitamin D status according to region of the world. Six regions of the world were reviewed - Asia, Europe, Middle East and Africa, Latin America, North America, and Oceania-through a survey of published literature. Based on the articles referred to in this review, it was concluded that insufficient vitamin D status is prevalent in every of the six regions studied. Depending on the region, between 50% and more than 90% of people had 25(OH)D concentrations below 50 nmol/L. Low vitamin D status is most common in regions such as South Asia and the Middle East. Data demonstrate that insufficient vitamin D status is widespread and is re-emerging as a major health problem globally. Urbanization in combination with modern and also traditional lifestyles such as indoor working, indoor leisure time activities, and traditional Islamic clothing, and in combination with the aging process (institutionalization) is an important risk factor for vitamin D insufficiency/deficiency in large parts of the adult population. In highly urbanized areas, individual daily sun exposure is usually too low to achieve a 25(OH)D level of 75 nmol/L. Due to the fact that the vast majority of foods naturally contain no or only modest amounts of vitamin D, diet is not able to close the gap in vitamin D supply. It is noteworthy that urbanization and industrialization has long been known as a major cause of childhood rickets in western countries [7]. Rickets is now on the increase in many developing countries, and is also re-emerging as an important health problem in countries with strong sun avoidance policies and cultures requiring modest dress. 

## 4. Diseases Associated with Nonclassical Vitamin D Actions

**Figure 1:**
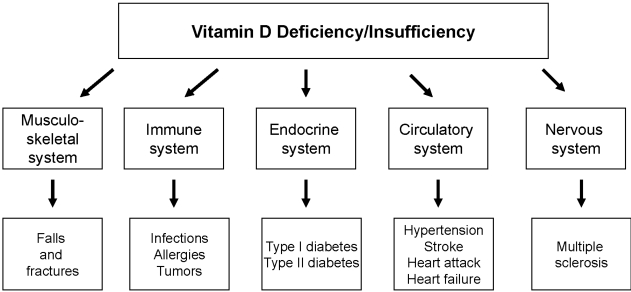
Suggested association of vitamin D deficiency/insufficiency with chronic diseases.

Figure 1 illustrates that vitamin D deficiency/insufficiency can result in impaired musculo-skeletal function, impaired immune function, cardiac and vascular impairment and impaired nervous function. As outlined in Figure 1, the development of various chronic diseases may be the consequence.

### 4.1. Vitamin D and Muscle Strengthening

Vitamin D deficiency causes reduced aktomyosin content of myofibrils, low calcium content of mitochondria, reduced calcium uptake into the sarcoplasmic reticulum, and low serum levels of muscle enzymes [3]. The importance of vitamin D-repletion for adequate muscle function was underscored in a recent study in institutionalized people ≥60 years of age with insufficient vitamin D status [20]: This RCT demonstrated that six-month supplementation (December to May) of oral vitamin D (3,750 µg once a month during the first two months, followed by 2,250 µg once a month for the last four months) was able to improve lower limb muscle strength by 16-24%. Data support results of a recently performed meta-analysis of randomized controlled trials (RCTs), indicating that daily doses of 17.5 to 20 µg supplemental vitamin D are able to prevent falls in elderly adults [21]. The relative risk of falls was reduced by approximately 20% if the achieved serum 25(OH)D concentrations is 60 nmol/l or more. In contrast to “high dose” supplemental vitamin D, low dose daily supplemental vitamin D (5 to 15 µg) is not able to prevent falls. Thus, doses of supplemental vitamin D of less than 17.5 µg or serum 25-hydroxyvitamin D concentrations of less than 60 nmol/L may not reduce the risk of falling among older individuals. It is noteworthy that in elderly people the risk of falling predicts the risk of developing osteoporotic fractures. Therefore, the effects of vitamin D on muscle strength may contribute to the preventive effect of vitamin D on osteoporotic fractures. There is also evidence that adequate vitamin D supply is important for muscle function in children. Already more than 50 years ago, Ronge [22] has demonstrated that children who have hands and face exposed to UVB radiation in their classroom at school for 3-5 hours during wintertime show better endurance performance compared to a control group without UVB exposure. Endurance performance was assessed by bicycle ergometry. In that study, a similar positive effect on endurance performance was seen in children who received a single vitamin D bolus of 6.25 mg vitamin D in February. 

### 4.2. Infections

There is mounting evidence for a pivotal role of vitamin D in the immune system. Calcitriol is able to induce the differentiation of monocytes into macrophages. In addition, calcitriol increases the activity of macrophages and facilitates their cytotoxic activity. Macrophages represent the first unspecific defence line of the immune system. It is well known that the prevalence of infections such as pneumonia is high in infants with rickets [3]. The use of vitamin D (or cod liver oil) as a treatment of infections have been practised for over 150 years. As early as 1903, Niels Finsen was awarded the Nobel Prize for Medicine and Physiology for his theory to cure Lupus vulgaris (skin-tuberculosis) using phototherapy. In 2007, Schauber *et al.* [23] published data demonstrating that vitamin D is able to stimulate synthesis of the anti-microbial peptide cathelicidin in human skin cells to enhance innate immunity. A meta-analysis of observational studies has demonstrated that patients with tuberculosis have lower circulating 25(OH)D concentrations compared to healthy controls [24]. Ecological studies also support a preventive role of vitamin D in influenza: the seasonal and latitudinal distribution of outbreaks of influenza A in the world in 1967-1975, and weekly consultation rates for illnesses diagnosed clinically as influenza or influenza-like in England 1968-1970 were inversely associated with solar UVB radiation [25]. Very recently, it has been demonstrated in an RCT that supplementation with 30 µg vitamin D daily reduces the risk of wintertime influenza A in Japanese nursery school children [26]. Some epidemiological data support the assumption that vitamin D may reduce the susceptibility to respiratory tract infections [27,28]. In addition, vitamin D users of the RECORD trial [29], an RCT with approximately 3,500 participants who received 20 µg vitamin D or placebo, reported a lower tendency for infections and antibiotic use in March compared to vitamin D nonusers. In another RCT in individuals with baseline circulating concentrations below 50 nmol/L, supplementation with 20 µg or 50 µg vitamin D daily for three years significantly reduced upper respiratory tract infections compared to placebo [30]. In contrast, a daily vitamin D supplement of 50 µg for 12 weeks did not prevent upper respiratory tract infections in individuals with baseline circulating 25(OH)D concentrations above 50 nmol/L [31]. Consequently, there is currently insufficient data to conclusively state that vitamin D supplementation could result in lowered infection [32]. One factor that has to be considered in future studies is baseline 25(OH)D concentration. In addition, the relation between vitamin D supplementation, local calcitriol, and local cathelicidin production has to be investigated more detailed. Interestingly, oral intake of activated vitamin D in rickets patients for four weeks significantly increased human cathelicidin expression in neutrophils compared to age-matched healthy controls without administration of activated vitamin D [33], indicating a critical role of adequate calcitriol availability for regulation of the innate immune response. 

### 4.3. Allergies

Activation of the adaptive immune system is complex. Generally, it is of importance that specific pathways of the specific immune system are adequately suppressed in order to avoid autoimmune diseases or allergic reactions. Regulatory T cells are crucial for the maintenance of immunological tolerance. Their major role is to shut down T cell-mediated immunity toward the end of an immune reaction and to suppress auto-reactive T cells. A strong Th2 predominance leads to pathologic conditions such as overproduction of IgE and allergic diseases, whereas a strong Th1 predominance leads to autoimmunity and severe allograft rejection. Of clinical importance is the fact that DCs may induce naïve T cells in an immunogenetic direction but also in a tolerogenic direction, depending on the state of their maturation and their cell surface receptor. Tolerogenic DCs generally are semimature. There is accumulating evidence that vitamin D modulates the adaptive immune system [16]. Calcitriol appears to generate tolerogenic DCs *in vivo*, as demonstrated in models of transplantation and autoimmune disease. DCs appear to be key targets of calcitriol. Calcitriol arrest the differentiation and maturation of DCs, maintaining them in an immature state. Calcitriol is able to enhance the secretion by DCs of the anti-inflammatory and anti-allergic cytokine IL-10. 

At present, the vitamin D hypothesis of allergies takes two forms: Some argue that vitamin D deficiency may cause allergic reactions whereas others argue that vitamin D excess leads to an increased allergy risk. Wjst is a representative of the latter hypothesis. He argues that the increase in allergies in Bavaria after 1960 coincided with vitamin D supplementation intervention programs to prevent rickets in childhood. Moreover, both, adherence to these programs and prevalence of allergies in children seem to be lower in farming communities in Bavaria [34]. The farm protection is observed mainly during the first year of life [35], when vitamin D supplementation is also recommended. Wjst’s hypothesis is based on the assumption that vitamin D may lead to Th2 predominance and increased IgE production. Generally, his hypothesis is supported by findings that children whose mothers' concentration of 25(OH)-vitamin D in late pregnancy was >75 nmol/l had an increased risk of eczema on examination at nine months and asthma at age nine years compared to children whose mothers' concentration was <30 nmol/L [36]. In addition, vitamin D supplementation during infancy was associated with a higher allergy risk [37,38], and the prevalence of allergic rhinitis increased across quartile groups of 25(OH)D serum levels in adults of NHANES III [39]. 

It is, however, noteworthy that several other epidemiological studies support the vitamin D deficiency hypothesis of allergic reactions [40,41,42,43,44]. Moreover, administration of calcitriol to blood cells of healthy persons and steroid-resistant asthmatic patients enhanced subsequent responsiveness to dexamethasone for induction of IL-10 [43]. Very few intervention trials are available so far. In a small, randomized, double-blind, placebo-controlled trial, vitamin D_2 _supplementation (25 µg/day) significantly improved skin symptoms in children with winter-related atopic dermatitis [45]. In a study in heart failure patients, vitamin D_3 _supplementation (50 µg/day) was able to increase blood levels of the anti-allergic cytokine IL-10 [46]. However, the effect on allergic reactions has not been elucidated in that earlier investigation. 

In total, it cannot be ruled out that vitamin D deficiency as well as vitamin D excess may increase the risk of allergic reactions. This assumption is supported by recent findings. Hyppönen *et al.* [47] observed a biphasic effect of vitamin D with both low and high 25(OH)D levels associated with elevated IgE concentrations in participants of the 1958 British birth cohort. Compared with the reference group with the lowest IgE concentrations [25(OH)D 100-125 nmol/L], adjusted IgE concentrations were 29% higher for participants with the 25(OH)D < 25 nmol/L, and 56% higher for participants with 25(OH)D > 135 nmol/L. 

### 4.4. Cancer

Since vitamin D is a key regulator of various cellular metabolic pathways, it is important for cellular maturation, differentiation, and apoptosis [3]. In 2008, the WHO published a report from the International Agency for Research on cancer [48] that came to the conclusion that there is (i) consistent epidemiological evidence for an inverse association between 25(OH)D and colorectal cancer and colorectal adenomas, (ii) suggested epidemiological evidence for an inverse association between 25(OH)D and breast cancer, (iii) insufficient evidence for an inverse association between 25(OH)D and other types of cancer, and (iv) the need for new randomized controlled trials (RCTs). One such RCT has already been published [49]: In a four-year, population-based study, where the primary outcome was fracture incidence, and the principal secondary outcome was cancer incidence, 1179 community-dwelling women were randomly assigned to receive 1500 mg supplemental calcium/d alone (Ca-only), supplemental calcium plus 27.5 µg vitamin D/d (Ca + D), or placebo. Cancer incidence was 60-77% lower in the Ca + D women and 43% lower in the Ca-only group than in the placebo control subjects (P < 0.03). Gorham *et al.* [50] have estimated that in North America, Europe, and East Asia approximately 32% of colon cancer and approximately 26% of breast cancer can be prevented with 50 µg vitamin D daily and 3-10 min daily of noon sunlight seasonality, when weather permits. Garland *et al.* [51] estimated that raising the minimum year-around serum 25(OH)D level to 100-150 nmol/L would prevent approximately 58,000 new cases of breast cancer and 49,000 new cases of colorectal cancer each year, and three fourths of deaths from these diseases in the United States and Canada. Such intakes also are expected to reduce case-fatality rates of patients who have breast, colorectal, or prostate cancer by half. Nevertheless, there is also some concern that cancer risk is not only enhanced in individuals with deficient/insufficient vitamin D status, but also if 25(OH)D concentrations rise above 80 nmol/L [52], a concentration several vitamin D researchers consider adequate. However, this increase in cancer risk has only been observed in observational studies after multivariable adjustments have been made for confounding factors. This kind of exploratory data analysis has been criticized by some researchers [53]. 

### 4.5. Diabetes Mellitus

*In vitro* and *in vivo* studies suggest that vitamin D can prevent pancreatic beta-cell destruction and reduces the incidence of autoimmune diabetes. This may at least in part be due to a suppression of proinflammatory cytokines such as tumor necrosis factor (TNF)-α. Recently, the relationship between UVB irradiance, the primary source of circulating vitamin D in humans, and age-standardized incidence rates of type 1 diabetes mellitus in children aged <14 years, was analyzed according to 51 regions of the world [54]. Incidence rates were generally higher at higher latitudes and were inversely associated with UVB irradiance. As early as 2001, Hyppönen *et al.* [55] has demonstrated in a birth cohort study that vitamin D supplementation was associated with a decreased frequency of type 1 diabetes. In contrast, children suspected of having rickets during the first year of life had a three times higher relative risk compared with those without such a suspicion. Meanwhile, a meta-analysis of four case-control studies has shown that the risk of type 1 diabetes is reduced by 29% in infants who are supplemented with vitamin D compared to those who are not supplemented [56]. There is also some evidence of a dose-response effect, with those using higher amounts of vitamin D being at lower risk of developing type 1 diabetes. Finally, timing of supplementation might also be important for the subsequent development of type 1 diabetes. In a recent RCT [57], the majority of patients with latent autoimmune diabetes in adults increased their concentrations of plasma C-peptide levels in fasting state after 1 year of treatment with activated vitamin D, whereas only a minority of patients treated with insulin alone maintained stable fasting C-peptide levels. 

In 2007, Pittas *et al.* [58] conducted a systemic review and meta-analysis for observational studies and clinical trials in adults with outcomes related to glucose homeostasis in type 2 diabetes mellitus. Observational studies show a relatively consistent association between low vitamin D status and prevalent type 2 diabetes, with an odds ratio of 0.36 among non-Blacks for highest *versus* lowest 25-hydroxyvitamin D. Evidence from RCTs with vitamin D and/or calcium supplementation suggests that combined vitamin D and calcium supplementation may have a role in the prevention of type 2 diabetes only in populations at high risk (*i.e,.* glucose intolerance). Whereas vitamin D supplementation did not improve glycemic control in diabetic subjects with normal serum 25(OH)D levels [59], administration of 100 µg vitamin D3 improved insulin sensitivity in vitamin D deficient and insulin resistant South Asian women [60]. Insulin resistance was most improved when endpoint serum 25(OH)D reached ≥ 80 nmol/L. Optimal vitamin D concentrations for reducing insulin resistance were shown to be 80-119 nmol/L. 

### 4.6. Cardiovascular Disease

Globally, cardiovascular disease (CVD) is the number one cause of death. In 2005, CVD was responsible for approximately 30% of deaths worldwide. CVD includes various illnesses such as coronary heart disease (CHD), peripheral arterial disease, cerebrovascular disease such as stroke, and congestive heart failure. There is accumulating evidence that the vitamin D hormone calcitriol exerts important physiological effects in cardiomyocytes, vascular smooth muscle cells, and the vascular endothelium. The mechanisms have been reviewed in detail elsewhere [61]. Hypertension is a key risk factor for CVD. A recently published systematic review and meta-analysis came to the conclusion that vitamin D produces a fall in systolic blood pressure of −6.18 mm Hg and a nonsignificant fall in diastolic blood pressure of −2.56 mm Hg in hypertensive patients. No reduction in blood pressure is seen in studies examining patients who are normotensive at baseline [62]. Since these studies had small sample sizes, future studies should investigate their generalizability. 

Several large prospective observational or cohort studies have demonstrated that a higher vitamin D status is associated with approximately 50% lower cardiovascular morbidity and mortality risk compared with low vitamin D status (Table 2). 

**Table 2:** Evidence for association of circulating 25-hydroxyvitam in D level with cardiovascular morbidity and mortality.

Study	Design	Number of individuals	Comparator	Odds/hazard ratio or Relative risk (95% CI)
**Fatal stroke**				
Pilz *et al.* 2009 [63]	Prospective cohort study with coronary angiography	3258	Per z value of 25(OH)D	OR 0.58 (0.43 to 0.78)
**Cardiovascular **				
**morbidity**				
Wang *et al.* 2008 [64]	Prospective observational study	1739	25(OH)D > 37.5 nmol/L *versus *< 25 nmol/L	HR 0.55 (0.32 to 0.97)
**Myocardial **				
**infarction**				
Giovannucci *et al.* 2008 [65]	Nested case control study	1354	25(OH)D > 75 nmol/L *versus* < 37.5 nmol/L	RR 0.48 (0.28 to 0.81)
**Cardiovascular **				
**mortality**				
Dobnig *et al.* 2008 [66]	Prospective cohort study with coronary angiography	3258	Median 25(OH)D 70 nmol/L *versus* 19 nmol/L	HR 0.45 (0.32 to 0.64)
Pilz *et al.* 2009 [67]	Prospective observational study in individuals 50-75 years	614	Three highest *versus* lowest 25 (OH)D quartile	HR 0.19 (0.07 to 0.50)
Ginde *et al.* 2009 [68]	Prospective observational study in individuals > 65 years.	3408	25(OH)D > 100 nmol/L *versus* < 25 nmol/L	HR 0.42 (0.21 to 0.86)

The Women’s Health Initiative (WHI) calcium/vitamin D (CaD) trial could however not demonstrate a reduction in cardiovascular mortality by daily supplementation of 1,000 mg calcium and 10 µg vitamin D [69]. Meanwhile it is clear that an amount of 10 µg vitamin D is far too low to result in a meaningful increase in serum 25(OH)D levels (see before) and that a daily calcium supplement of 1,000 mg increases the risk for cardiovascular events in healthy older women. Both, the supplemental calcium in the vitamin D arm of the WHI study and the low amount of vitamin D might have countermanded its cardiovascular benefits. In line with this assumption, a recent meta-analysis of seven randomized trials showed a slight but statistically nonsignificant reduction in CVD risk (relative risk: 0,90; 95% CI: 0.77 to 1.05) with vitamin D supplementation at moderate to high doses (approximately 25µg/d) but not with calcium supplementation (relative risk: 1,14; 95% CI: 0.92 to 1.41) or a combination of vitamin D and calcium supplementation (relative risk: 1.04; 95% CI: 0.92 to 1.18) [70]. 

In line with a potential beneficial effect of vitamin D on CVD risk, a daily vitamin D supplement of 83 µg could improve some traditional and nontraditional cardiovascular risk markers in healthy overweight and obese subjects with mean 25(OH)D concentrations of 30 nmol/L who attended a weight-reduction program [71].

### 4.7. Multiple Sclerosis

Multiple sclerosis (MS) is a demyellinating disease of the central nervous system that is debilitating and can be fatal. Manifestation of the disease is typically between the age of 20 and 40. In Europe and North America, regions with higher UVB radiation have low rates of MS and vice versa [3]. In Israel, MS prevalence depends on the country of origin. The prevalence is high in people who were born in a country with low UVB irradiance [72], indicating that vitamin D status during the period of early life is of importance for MS susceptibility. MS disease activity shows inverse fluctuations according to season and vitamin D status [73]. In a prospective, nested case-control study among more than seven million US military personnel [74], MS prevalence was lower in those people who had circulating 25-hydroxyvitamin D concentrations between 100 and 150 nmol/L compared with those who had 25-hydroxyvitamin D concentrations below 63 nmol/L. However, this association was only seen in Whites and not in Blacks, indicating that genetic factors play an important role in the pathogenesis of MS. Therefore, the recent finding is of importance that expression of the MS-associated MHC class II allele HLA-DRB1*1501 is regulated by Vitamin D [75]. 

## 5. Mortality

As mentioned before, vitamin D status is an important independent predictor of CVD and specific types of cancer. In addition, vitamin D status predicts CVD and cancer mortality. Both, CVD and cancer are the most important causes of mortality in developed countries. In 2007, Autier and Gandini [76] published a meta-analysis of randomized controlled trials (RCTs) on vitamin D and mortality that were not primarily designed to assess mortality. The authors found out that in middle-aged and elderly patients with low serum concentrations of 25-hydroxyvitamin D (25(OH)D) vitamin D supplementation was linked to lower all-cause mortality compared to no vitamin D supplementation. Daily dose of vitamin D ranged between 10 µg and 50 µg. Risk reduction was 7% during a mean follow-up of 5.7 years.

Based on the aforementioned meta-analysis, several large prospective cohort studies were recently published on all-cause mortality and vitamin D status (Table 3). They demonstrate a consistent increase in mortality risk in patients with insufficient or deficient 25(OH)D concentrations. However, low 25(OH)D was not an independent predictor for mortality in patients with advanced disease [77,78]. One may speculate that in this case, vitamin D supplementation is unable to reverse the already existing severe pathophysiologic derangements. 

**Table 3:** Evidence for association of circulating 25-hydroxyvitamin D level or vitamin D supplementation with all-cause mortality.

Study	Design	Number of individuals	Comparator	Hazard ratio or relative risk (95% CI)
Autier and Gandini, 2007 [76]	Meta-analysis of 18 vitamin D supplementation studies	57,311	Supplemented *versus* unsupplemented	RR 0.93 (0.87 to 0.99)
Dobnig *et al.* 2008 [66]	Prospective cohort study with coronary angiography	3,258	Median 25(OH)D 70 nmol/L *versus* 19 nmol/L	HR 0.48 (0.37 to 0.63)
Kuroda *et al.* 2009 [77]	Prospective observational study in postmenopausal women	1,232	≥ 50 nmol/L *versus *< 50 nmol/L	HR 0.46 (0.27 to 0.79)
Ng *et al.* 2008 [78]	Prospective cohort study in patients with colorectal cancer	304	Mean 41 nmol/L *versus* 100 nmol/L	HR 0.52 (0.29 to 0.94)
Ginde *et al.* 2009 [68]	Prospective observational study in individuals > 65 years.	3,408	25(OH)D > 100 nmol/L *versus* < 25 nmol/L	HR 0.55 (0.34to 0.88)
Pilz *et al.* 2009 [67]	Prospective observational study In individuals 50-75 years	614	Three highest quartiles *versus* lowest quartile	HR 0.51 (0.28 to 0.93)

## 6. Conclusions

In 2003, a review article had summarized the association of insufficient vitamin D status with various diseases such as myopathy, CVD, cancer, diabetes mellitus, MS, and infections [8]. Meanwhile, evidence has accumulated that vitamin D may indeed play an important role in the etiology of many of these diseases. Meta-analyses of RCTs demonstrate that vitamin D improves muscle function and seems to reduce blood pressure in hypertensive patients. In addition, some RCTs demonstrate that vitamin D reduces cancer incidence, and improves glucose homeostasis in patients with type 2 diabetes and cardiovascular risk markers in overweight people [49,60,71]. The most exiting result is however the fact that vitamin D may reduce mortality rate. This latter finding fits well together with the fact that severe deficiency of several other vitamins such as retinol, thiamine, niacin, and ascorbic acid is also associated with enhanced mortality. Nevertheless, additional large RCTs are needed to confirm whether or not vitamin D is able prolong survival in individuals with inadequate vitamin D status. In this context, the effect of vitamin D in deficient and insufficient individuals should be investigated separately. 

Some aforementioned beneficial data on glucose homeostasis and cardiovascular risk markers were not confirmed by recent RCTs [59,81]. All these RCTs performed so far were relative small in sample seize [59,60,71,81]. In addition, individual medication and baseline circulating 25(OH)D concentrations may have influenced study results. Therefore, additional research is necessary to clarify whether or not vitamin D supplementation is indeed effective in secondary prevention and also in tertiary prevention of chronic diseases. But we should be aware of the fact that many chronic diseases are of multi-factorial origin. Vitamin D is certainly only one factor among others. In addition, there may be individual differences with respect to the metabolic pathways that are disturbed in vitamin D deficient persons. Therefore, we should not be too enthusiastic that future RCTs will show clear beneficial vitamin D effects. For example, the meta-analysis by Autier and Gandini was based on more than 55,000 individuals. None of the single studies included in this analysis showed a significant vitamin D effect on mortality, indicating that huge sample seizes are probably needed to demonstrate a clear vitamin D effect. Even so, the consequences on a population scale may be important because of the large number of people who are affected. 

The effect of vitamin D on MS, type 1 diabetes, infections, and allergies is less clear at present. Although newborns usually receive vitamin D supplements for preventing rickets, possible adverse effects of deficient vitamin D concentrations during fetal development such as increased susceptibility for type I diabetes and MS have to be considered as well. It is noteworthy that many women of childbearing age worldwide are vitamin D insufficient or even deficient [19,82]. With respect to MS, type 1 diabetes, and allergies, more birth cohort studies are needed. 

Despite some uncertainties with respect to vitamin D and health, there is general agreement that currently a high percentage of people worldwide have low vitamin D status [19,83]. The recommended daily vitamin D intake of 5-15 µg is too low to achieve an adequate vitamin D status in people with only modest UVB exposure. Generally, treating vitamin D deficiency is easy to perform, safe, and inexpensive. Sources of vitamin D could include a combination of food fortification, supplements, and natural and artificial UV-B irradiation, if properly acquired. It has been calculated that 1 µg vitamin D increases circulating 25(OH)D levels by approximately 1 nmol/L [4]. Thus, a daily intake of approximately 50 µg vitamin D would be necessary for increasing the circulating 25(OH)D level from 25 nmol/L to 75 nmol/L. In order to achieve a 25(OH)D concentration above 75 nmol/L in almost all individuals of a group with mean baseline 25(OH)D concentrations of 38 nmol/L, daily supplementation with up to 100 µg vitamin D is necessary [5]. In otherwise healthy adults, the risk of vitamin D intoxication is extremely rare [3,84]. Vitamin D intoxications such as hypercalcemia do not occur until oral vitamin D intake and serum 25(OH)D concentrations exceed 250 µg/day (approximately 3-5 µg/kg body weight) [84] and 372 nmol/L [6], respectively. A daily amount of up to 250 µg vitamin D is similar to the amount that is produced by daily whole body exposure to UVB radiation [10]. 
